# The efficacy and potential mechanisms of pyrotinib in targeting EGFR and HER2 in advanced oral squamous cell carcinoma

**DOI:** 10.1186/s12903-024-04459-4

**Published:** 2024-08-06

**Authors:** Liang Zhou, Kehao Le, Qianming Chen, Huiming Wang

**Affiliations:** 1 Head and Neck Surgery, the Affiliated Sir Run Run Shaw Hospital, Zhejiang University School of Medicine, Hangzhou, 310016 PR China; 2 Oral and Maxillofacial Surgery, the Stomatology Hospital, Zhejiang University School of Medicine, No.166 Qiutao Road, Hangzhou, 310016 Zhejiang PR China

**Keywords:** Advanced OSCC, Pyrotinib, EGFR, HER2

## Abstract

**Background:**

Human epidermal growth factor receptor 2 (HER2) plays an important role in the progression of multiple solid tumors and induces resistance to epidermal growth factor receptor (EGFR) target treatment. However, the expression status and the clinical significance of HER2 in oral squamous cell carcinoma (OSCC) is still controversial. Pyrotinib (PYR) is a promising novel EGFR/HER2 dual inhibitor, whose efficacy in OSCC has not been determined.

**Methods:**

57 locally advanced de novo OSCC patients were included in this study to investigate the relationship between the HER2 expression levels and the prognosis by the tissue microarray analysis (TMA). In vitro and in vivo experiments were performed to retrieve the efficacy of PYR in OSCC. The main downstream of HER2 was evaluated by western blotting in OSCC cell lines and xenograft tumors to explore the potential mechanism of PYR.

**Results:**

This study revealed the primary tumor of OSCC had higher HER2 expression levels. Patients with HER2 overexpression had poor overall survival (*P* < 0.014) and poor disease free survival (*P* < 0.042). In vitro, PYR suppressed the proliferation, colony formation and migration of OSCC cells. It also promoted apoptosis of OSCC cells and induced cell cycle arrest. Furthermore, PYR was able to inhibit the occurrence and development of OSCC effectively in vivo. Western blotting revealed that PYR suppressed OSCC by inhibiting the phosphorylation of HER2, AKT and ERK.

**Conclusions:**

This study exhibited the anti-OSCC effects of PYR in vitro and in vivo, and demonstrated PYR inhibited OSCC cells by inducing apoptosis via the HER2/ AKT and ERK pathway. The result of this study also indicated locally advanced OSCC patients might benefit from HER2 assay and EGFR/HER2 dual inhibit treatment.

**Supplementary Information:**

The online version contains supplementary material available at 10.1186/s12903-024-04459-4.

## Background

Oral squamous cell carcinoma (OSCC), classically presents with a non-healing mouth sore or ulcer, is the most common head neck squamous cell carcinoma (HNSCC) with 350,000 new cases and 170,000 deaths per year [1, 2]. The diagnosis of OSCC must be established by a pathology examination, of which the histopathological spectrum is characterized by the extent of cellular atypia and squamous differentiation [3]. The principal modalities of curative therapy of OSCC are surgery, radiation, systemic therapy, et al [3, 4]. Target therapy, such as varies tyrosine kinase inhibitor (TKI), has been the important component of systemic therapy in the clinical practice. As a novel oral, irreversible pan-ERBB TKI it is, pyrotinib (PYR) has demonstrated a promising single-agent efficacy and acceptable tolerability in cancer treatment [5, 6].

Although the early stages of OSCC have gained good prognosis despite degraded neck dissection extent [7, 8], the treatment of advanced OSCC is still full of challenges, leading to the poor survival outcomes [9, 10]. In addition to the surgery and traditional adjuvant therapy, the epidermal growth factor receptor (EGFR) target therapy provides survival benefits to advanced OSCC patients and is recommended in the National Comprehensive Cancer Network (NCCN) guideline [11]. Until now, the Food and Drug Administration (FDA) only approved one EGFR target drug for clinical practice, which is cetuximab. However intrinsic and acquired resistance to cetuximab is frequently encountered in clinics [12]. A novel biomarker for predicting poor prognosis of OSCC and targeting individualized drug delivery is urgently needed to overcome the dilemma, and is the focus of many clinical research studies.

Both HER2 and EGFR belong to the ERBB tyrosine kinases receptor family. The overexpression of HER2 forms heterodimerization with the other ERBB members, leading to the initiation of various signaling pathways by auto-phosphorylating tyrosine residues, and inducing resistance to EGFR target therapy [13]. Multiple clinical trials focusing on HER2-targeted therapies have resulted in exciting outcomes in the treatment of various solid tumors with abnormal expressions of HER2 [14–16]. As a robust irreversible EGFR/HER2 dual TKI, PYR has shown remarkable responses in many advanced cancers even with previously failed EGFR/HER2 targeted treatments [17, 18]. Therefore, it is worth exploring whether PYR has the equivalent efficacy in advanced OSCC.

By evaluating the HER2 clinical expression, this study aims to assess the possibility of the application of PYR in OSCC, and to investigate its anti-tumor effects, as well as the potential mechanisms in vitro and in vivo.

## Materials and methods

### Clinical samples and data

With the approval of the hospital’s ethics committee (keyan20210615-38, Figure S1), we retrospectively enrolled locally advanced OSCC patients with integrated clinical pathological information who accepted the initial curative surgery and standard adjuvant therapy at a tertiary referral hospital between January 2016 and June 2019. All of the included patients signed informed consent for the use of their tissues in this study. The inclusion criteria were de novo stage III or IVa OSCC patients with at least 2 years of follow up or with definitive survival outcome (death or recurrence). Formalin Paraffin Embedded blocks of primary tumor tissues and matched para-tumor normal oral mucosa tissues were used to generate tissue microarray (TMA) for the HER2 immunohistochemical (IHC) staining by an experienced pathologist. These blocks of tumor tissue were selected by the paired hematoxylin-eosin (H&E) stained sections, to confirm the presence of at least 80% tumor cells. Overall survival (OS) and disease free survival (DFS) were regarded as the main outcome variable.

### Cell lines, cell culture, reagents and animals

CAL27 (human OSCC cell line) and HN30 (human head neck squamous cell carcinoma cell line) were employed in the current study. Both of the cell lines were kindly provided by Professor Laiping Zhong (Shanghai Jiaotong University, school of medicine). CAL27 and HN30 cells were cultured in a growth medium consisting of 90% DMEM, 10% FBS and 1% antibiotics (penicillin and streptomycin). PYR was kindly provided by Jiangsu Hengrui Medicine Company (CHN.). All of the primary antibodies were purchased from Cell signaling technology (CST, USA.), including: anti-HER2 (4290&6942), phospho-AKT pathway antibody sampler kit (9916) and phospho-ERK1/2 pathway sampler kit (9911). 4-week-old, 20 to 22 g, male NOD-SCID gamma (NSG) mice were used for the xenografts under the approval of the ethics committee (SRRSH202107123).

### Cell proliferation assay

The cell proliferation ability of CAL27 and HN30 was assessed by cell viability and cell colony formation assay, and repeated three times. For cell viability assay, CAL27 and HN30 cells in a density of 1000 cells/well were routinely cultured in DMEM for 12 h in advance of PYR treatment. Then the cells were exposed to various concentrations of PYR or vehicles for 24, 48, or 72 h before undergoing cell counting kit-8 (CCK8, CK04, Dojindo, Japan) as the standard protocol. Survival curves were generated and the IC50 were calculated from the results of the CCK8 assay by GraphPad Prim 7.

For cell colony formation assay, CAL27 and HN30 cells in a density of 500 cell/well were seeded in DMEM for 24 h ahead of the treatment of PYR. These cells would be cultured under different concentrations of PYR for 2 to 3 weeks until obvious cell colonies formed. Cell colonies were then fixed and stained following the common methods. Only the colonies containing more than 50 cells were counted.

### Wound healing assay

CAL27 and HN30 were seeded and cultured until the cells grew to a confluent monolayer. A couple of parallel lines were made after starving the cells for 12 h. Then the cells were incubated under 10 μm/L PYR or vehicles for 12 h, and observed at hour 0, 6 and 12. The percentage of the wound closure area was used to reflect the cell migration ability, and calculated by Image J software.

### Apoptosis assay and cell cycle analysis

Through the treatment of 10 μm/L PYR, CAL27 and HN30 cells were harvested and fixed for apoptosis assay and cell cycle analysis as a standard protocol. After dual labeling by FITC-annexin V apoptosis detection kit (556,570, BD Bioscience, USA), flow cytometer and FlowJo software (FACSCalibur™, BD Bioscience, USA) were used to evaluate the apoptotic and necrotic OSCC cells following the operation manual. The cell cycle was labeled by PI/RNase (550,825, BD Pharmingen, San Diego, USA), and analyzed by flow cytometer. The expression levels of proteins related to the apoptosis signaling, including cleaved PARP, total caspase 3 and cleaved caspase 3 (9546, 9662,9661, CST, USA.), were examined by western blotting in CAL27 and HN30 cells with or without the treatment of PYR.

### Tumor xenografts

The in vivo study was conducted with the approvalof the hospital’s ethics committee (SRRSH202107123, Figure S2). CAL27 cells were harvested and resuspended at 2 × 10^6^ cells/ml cells with DMEM and Matrigel. Each seeded site wasinjected with a total volume of 0.1 ml CAL27 cells resuspension. A total of 8 NSG mice were randomly divided into the control group and the PYR group until the tumor xenografts grew to 100-150mm^3^. According to the groups, the mice were orally administered PYR solution (10 mg/Kg) or saline daily. The size of the tumors would be measured every other day, until the long diameter of each tumor wasapproaching 1.5 cm (the largest diameter of the tumors was 1.48 cm). Then the mice wereeuthanized by inhalation of 30% CO_2_ (4.5 L/min) for 2 minutes in a CO_2_ gas chamber, until they were motionless without breath or heart beating. After stopping the CO_2_, another 2 minutes observation was performed to confirm the death of the animals [19]. The tumors were harvested for the volume measurement, which was calculated by the following formula: V(tumor volume) = L(tumor length) × W^2^(tumor width) /2. Ki67 IHC assay was also applied to study the effect of PYR on the proliferation of the xenograft tumors [20].

### Western blotting

After the treatment of a gradient concentration PYR or control, CAL27 cells and CAL27 xenograft tumors were harvested for western blotting assay. The expression and phosphorylation level of the key protein in the HER2 cascade was assayed, including HER2, AKT, ERK. Following a standard protocol, total protein was extracted from the harvested samples. In order to ensure the equal loading, Bradford Assay kit (P0006C, Beyotime, CHN) was used to measure protein concentration. After dissolving the protein into SDS-PAGE, the samples were transferred onto a PVDF membrane. The primary antibodies were diluted at a 1:1000 dilution, while the fluorescently labeled anti-rabbit or anti-mouse IgG secondary antibodies (7076 and 7704, CST, USA) were diluted at a 1:10000 dilution. The internal control was established by Β-Actin (1226, CST, USA).

### Immunohistochemistry

IHC staining for HER2 and Ki67 was performed on the TMAs and the xenografts sections, according to standard protocol. A semiquantitative method was used to evaluate the IHC results by assigning a modified H-score to the TMAs samples [21]. The modified H-score was determined by multiplying the gradient percentage of positive cells per TMAs spot (0, 0 ~ 5%; 1,6 ~ 25%; 2, 26 ~ 50%; 3, 51 ~ 75%; 4, > 75%) by the staining intensity (0, negative or trace; 1, weak; 2, moderate; 3, intense). The protein expression was defined as high expression when the score was higher than the median score, or else it would be defined as low expression.

### Statistical analysis

All of the data was analyzed by SPSS23 (IBM, USA) and GraphPad Prism 7. Pearson’s chi-square test was used to analyze clinical data. The association of HER2 with T stage, Disease stage and Tumor site was determined by Fisher’s exact test. A paired t-test was applied to the paired data. an unpaired t-test or one-way ANOVA was applied according to the all other data category. When comparing two groups, the significance was determined using a student’s t-test. Comparison between multiple groups was determined by a one-way ANOVA test followed by Bonferroni post hot test. Statistically significant *P* values were indicated as *P* < 0.05. Survival curves were constructed using the Kaplan-Meier method, and the difference was assessed using the log-rank test [22]. Effect modifications were analyzed by univariate Cox regression analysis when the hazard ratio (HR) was significant (*P* < 0.05) [23]. The variables eventually selectedfor the multivariate model was determined by the results of an univariate Cox regression analyses and clinical evaluation.

## Results

HER2 expression was elevated in OSCC and positive correlated to poor survival.

In order to clarify the foundation of applying PYR in OSCC, a total of 57 locally advanced OSCC patients (44 males and 13 females) with the average age of 56.86 ± 9.7 years were finally included in this study for HER2 assay. The male patients were threefold more than the female in the present cohort, which was accordance with the epidemiological data [3]. While the mean age of the included patients in this study was approximate to some other OSCC or head neck cancer studies [7, 24, 25].

**Fig. 1:**
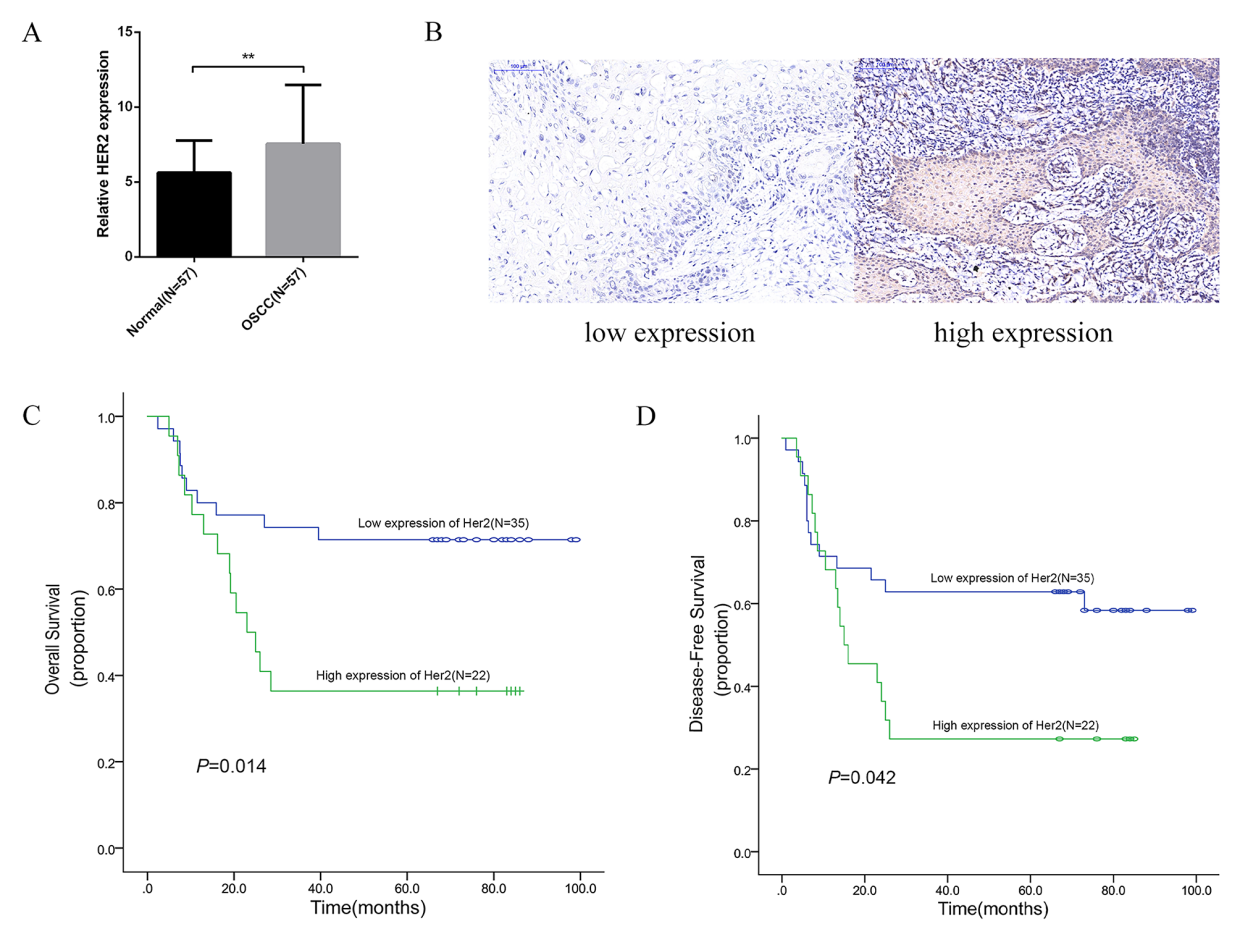
**A**. The expression levels of HER2 in the primary tumor tissue was significantly higher than the para-tumor mucosa tissue in the current cohort(7.56 ± 3.92 vs. 5.63 ± 2.14, *P* < 0.05) **B**: The low (left) and high expression (right) of HER2 in the primary tumors of OSCC in the TMA; **C-D**: Among 57 patients with locally advanced tumors, the OS and DFS of patients with high HER2 expression were significantly lower than those with low HER2 expression, and the difference was statistically significant

There were 47 stage IVa and 10 stage III patients with mean follow up of 51.65 ± 32.79 months (range: 2.4 ~ 99 months). Out of these patients, 30 patients suffered recurrence and 24 patients died during the follow up. The mean H-scores of HER2 expression in the tumor tissues was significantly higher than the scores in the para-tumor tissues (7.56 ± 3.92 vs. 5.63 ± 2.14, *P* < 0.05) (Fig. 1A).

Tumor tissues of 22 patients were assessed for high HER2 expression, while the others were assessed for low (Fig. 1B). There was no significant difference of the demographic and clinical pathological variables between the high and low HER2 expression groups (Table 1). While Kaplan-Meier analysis revealed both OS (41.7 ± 7.36 vs. 74.6 ± 6.61 m, *p* < 0.014) and DFS (33.1 ± 6.9 vs. 64.4 ± 7.33 m *p* < 0.042) were obvious shorter in the patients with high HER2 expression (Fig. 1C-D). The multivariate analysis was adjusted for potentially confounding clinical variables. The HER2 (categorical variable) also had an independent predictive ability for OS (*P* = 0.003), DFS (*P* = 0.016)(Table 2).

**Table 1:** Demographic and clinical data of all patients (*N* = 57)

	Total	Low	high	*P*
	(*N* = 57)	(*N* = 35)	(*N* = 22)	
Age (years)				0.587
Median	59	59	59	
Range	24–69	24–68	33–69	
< 60	30	17	13	
≥ 60	27	18	9	
Sex				0.532
Female	13	9	4	
Male	44	26	18	
Smoking status ^a^				0.953
Positive	36	22	14	
Negative	21	13	8	
Alcohol status ^b^				0.661
Positive	27	17	10	
Negative	30	18	12	
Tumor site				0.183
Tongue	25	14	11	
Gingiva	12	9	3	
Buccal	5	5	0	
Palate	8	3	5	
Floor of mouth	7	4	3	
T stage				0.534
T2	10	5	5	
T3	16	9	7	
T4	31	21	10	
N stage				0.935
N0	20	12	8	
N1	12	7	5	
N2	25	16	9	
Disease stage				0.136
III	10	4	6	
IV	47	31	16	

**Table 2:** Multivariate analysis of prognostic factors for OS and DFS of OSCC patients (*N* = 57)

Variables	OS					DFS										
	HR	95%CI		*P*					HR	95%CI			*P*			
TNM	2.554		0.716–9.114	0.149					1.611		0.591–4.388				0.351	
Smoke status	0.453		0.160–1.285	0.137				0.548				0.212–1.418		0.215		
Alcohol status	1.360		0.498–3.711	0.548				1.392				0.563–3.438		0.474		
PNI	4.128		1.182–14.408	**0.026**				3.097				1.079–8.891		**0.036**		
HER2	4.128		1.628–11.165	**0.003**				2.701				1.201–6.073		**0.016**		

Note: Boldface indicates *P* < 0.05. Variables were concluded as neither effect modifications nor confounding factors and not added into the multivariate Cox model

Abbreviations: OS, overall survival; DFS, disease-free survival

### PYR exhibfited the anti-proliferation and anti-migration effects on OSCC

**Fig. 2:**
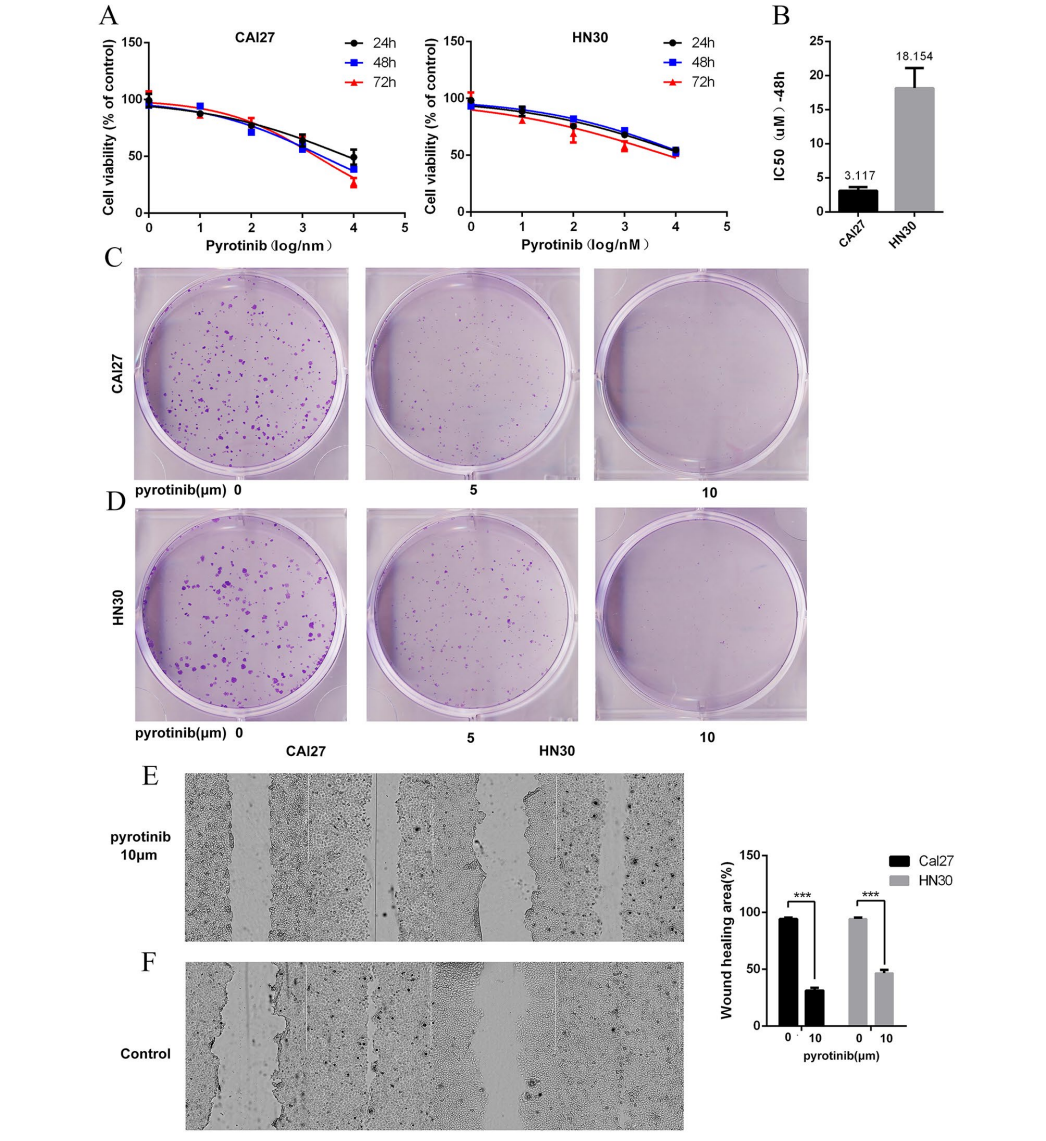
**A**: CCK8 assay was used to detect the toxicity and proliferation inhibition of PYR in OSCC cells **B**: It was found that PYR could effectively inhibit the proliferation activity of OSCC cells, with IC50 = 3.117 for CAL27 and 18.154 for HN30 at 48 h **C-D**: The effect of PYR on OSCC clone formation was investigated by monoclonal assay. 5 μm concentration effectively inhibited OSCC clone formation, while 10 μm concentration showed no obvious cell clone formation **E-F**: Cell scratch assay showed that 10 μm PYR could effectively decrease the migration of OSCC cells. After 24 h the cell migration ability was significantly different from that of the control group

To investigate whether PYR impaired the proliferation and migration of OSCC cells, cell viability, colony formation and wound healing assays were carried out subsequently in vitro. The result indicated that the viability of CAL27 and HN30 cells was inhibited by PYR in a concentration-dependent manner (Fig. 2A). The IC50(48 h) of PYR was 3.117 μm/L in CAL27 cells, and was 18.154 μm/L in HN30 cells respectively (Fig. 2B). PYR could significantly suppress the colony formation of CAL27 and HN30 cells in a similar concentration-dependent manner else. Contrasted to the negative control, nearly 80% number of cell colonies of CAL27 and HN30 were suppressed by PYR at the concentration of 5 μm/L, while over 90% colonies were inhibited at 10 μm/L (Fig. 2C-D). As shown in Fig. 2E-F, 10 μm/L of PYR apparently inhibited the cell migration of CAL27 and HN30 cells.

### PYR induced cell apoptosis and cell circle arrest in OSCC cell lines

**Fig. 3:**
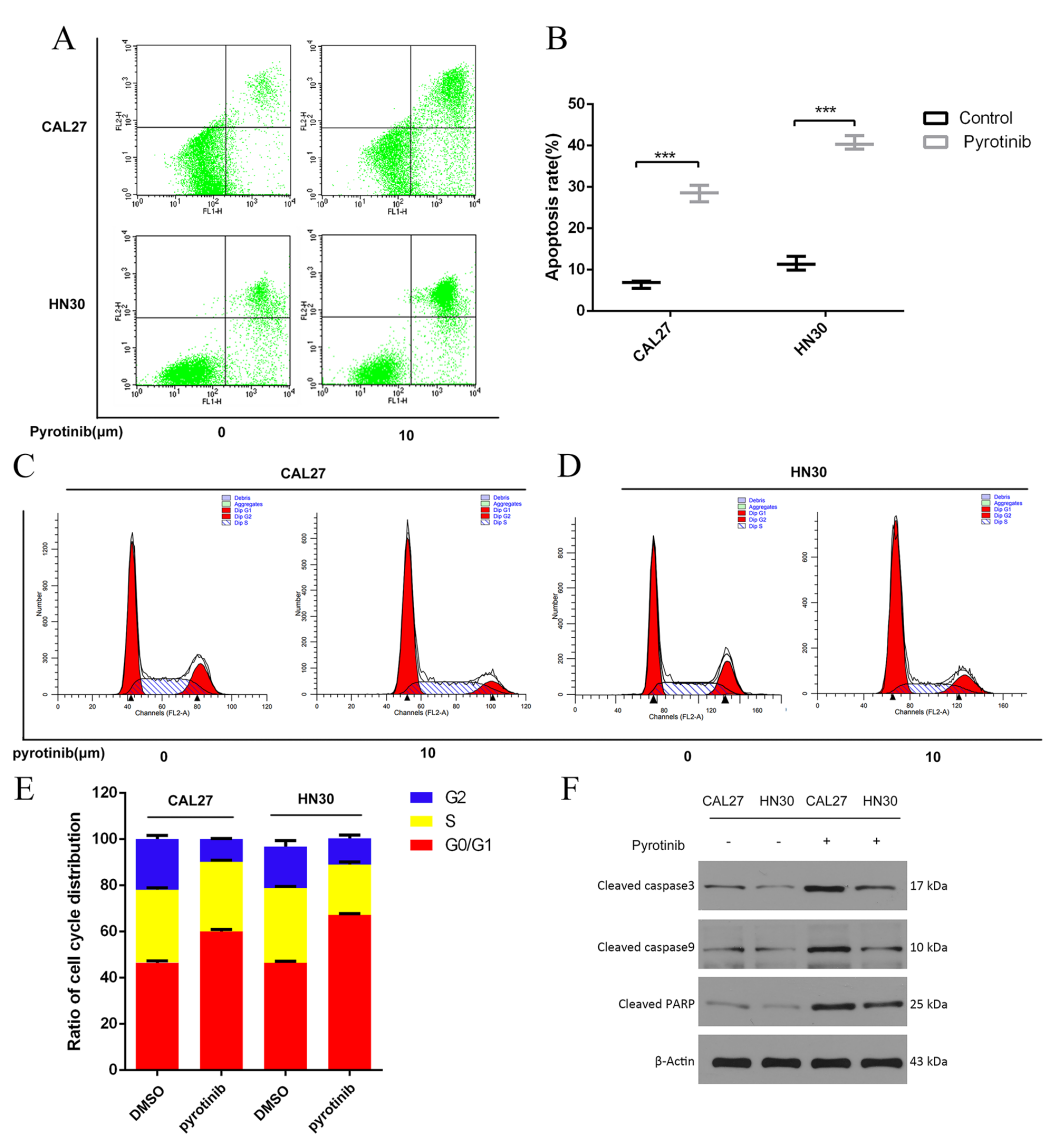
**A-B**: Flow cytometry showed that 10 μm PYR significantly increased the apoptosis of OSCC cells (CAL27 and HN30), compared with the control group **C-E**: Cell cycle detection showed that 10 μm PYR treated OSCC cells (CAL27 and HN30) significantly increased at G0/G1 phase, indicating that PYR can block OSCC cells at G0/G1 phase **F**: The expression levels of proteins related to the apoptosis signaling, including cleaved PARP, total caspase 3 and cleaved caspase 3, was examined by western blotting in CAL27 and HN30 cells treated under various concentrations of PYR for 24 h. The original gels are presented in Supplementary Figure S1

In the PYR group, the proportion of annexin-V-positive CAL27 cells ascended from 6.557 ± 0.522% to 28.477 ± 1.15%, while the proportion of the positive HN30 cells ascended from 11.49 ± 0.97% to 40.63 ± 0.96% (Fig. 3A-B). Flow cytometry assay also revealed PYR led to the cell cycle accumulation in G0/G1 phase, and decreased the approximate 10% proportion of G2/M and S phase cells (Fig. 3C-E). Meanwhile, the treatment of PYR dramatically increased the levels of cleaved caspase 3 and PARP, and decreased the levels of total caspase 3 in CAL27 and HN30 cells (Fig. 3F, Figure S3). Taking together, the result suggested significant cell apoptosis of CAL27 and HN30 cells was induced by PYR, as well as the G0/G1 cell circle arrest.

### PYR inhibited the tumor growth in vivo in CAL27 Xenograft models

**Fig. 4:**
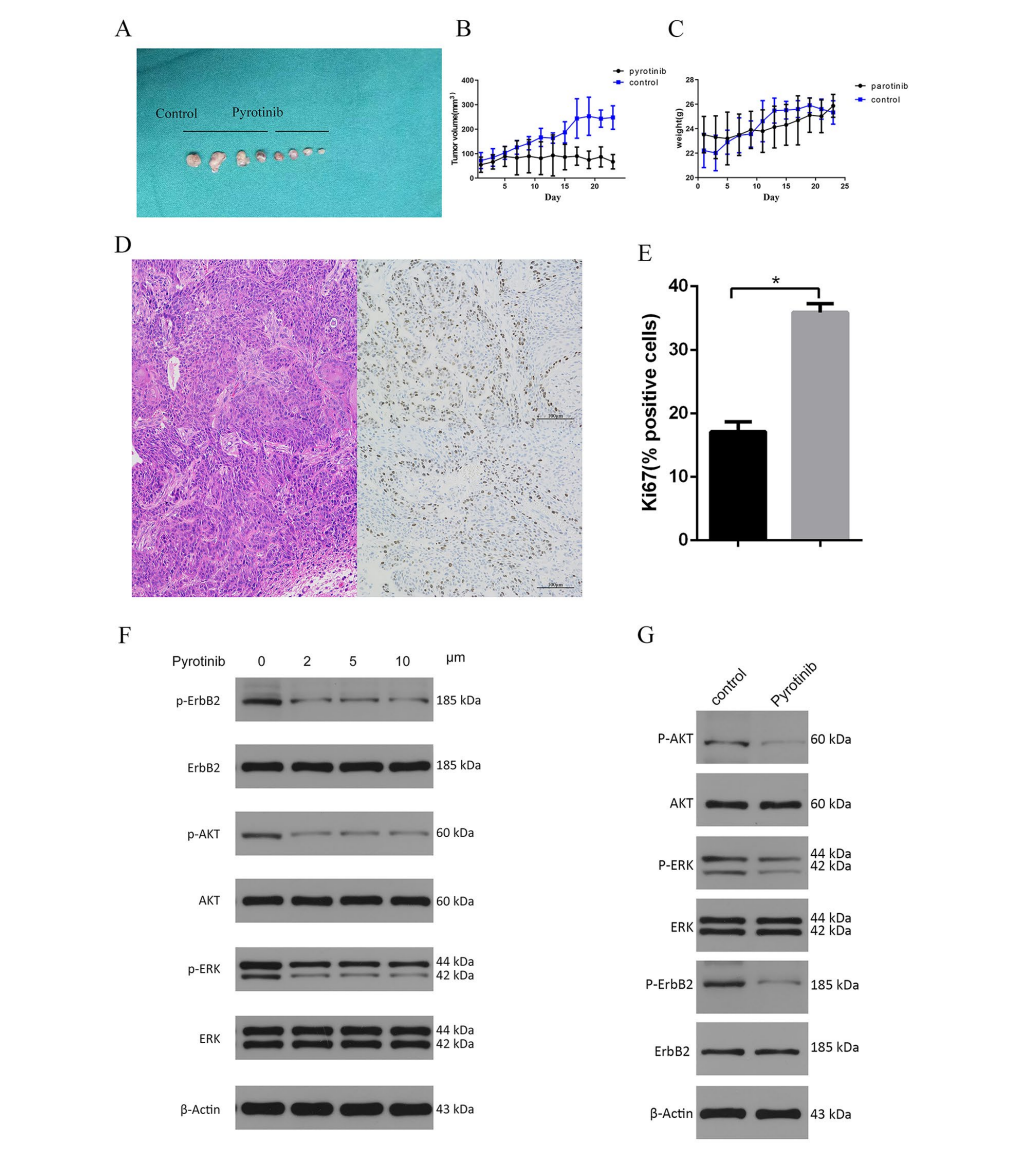
**A-C** In vivo xenograft experiments in nude mice showed that xenograft tumors in nude mice treated with PYR were significantly smaller than those in the control group, and there was no significant difference in the body weight of the mice **D-E** Immunohistochemical results of transplanted tumors HE and Ki67 showed that the proliferation activity of OSCC cells was significantly decreased after treatment with PYR (*P* < 0.05) **F-G**: Gradient concentration of PYR was used to treat CAL27 cells to detect the effect on the PI3K/AKT and MAPK signaling pathway. It was found that PYR could effectively inhibit the phosphorylation level of HER2 protein and the downstream of AKT/ERK, without significant changes in the total protein level. A similar result was observed in vivo as well. The original gels are presented in Supplementary Figure S2A-B

8 NSG mice xenograft models were successfully established by injecting CAL27 cells subcutaneously to determine whether PYR could inhibit tumor growth in vivo. The mean harvested tumor volume of the PYR group was significant smaller than the control group’s (67.08 ± 29.54 vs. 247.83 ± 48.00mm^3^, *P* < 0.01,Fig. 4A-B), while the body weight of the mice did not have a significant difference between the groups (Fig. 4C). Compared to the control group, the expression level of Ki67 of the harvested xenograft tumor was much lower in the PYR group, implying the inhibited tumor proliferation by PYR in vivo (Fig. 4D-E). In brief, PYR exhibited the robust anti-tumor effect and excellent bio-safety in vivo.

### The potential anti-tumor mechanism of PYR in OSCC

Since PI3K/AKT and mitogen-activating protein kinase (MAPK) signaling pathway was regarded as the main downstream of EGFR and HER2, western blotting therefore was employed to validated the effect of PYR in HER2 cascades in vitro and in vivo. Compared to the negative control, PYR dramatically downregulated the phosphorylation of HER2, AKT, and ERK, but did not influent the expression of these proteins themselves(Fig. 4F-G, Figure S4A-B). It suggested the anti-tumor mechanism of PYR in OSCC might be due to inhibit the PI3K/AKT and MAPK cascades by blocking the activation of HER2 protein.

## Discussion

The problem of EGFR target therapy resistance is particularly prominent in advanced OSCC, leading to poor prognosis [26, 27]. The overexpression of HER2 plays an important role in inducing resistance to the EGFR target therapy and promotes multiple tumor progression [28, 29]. A variety of HER2 or EGFR/HER2 dual target therapeutic strategies therefore are being explored to overcome the dilemma [30, 31]. Due to the lack of a well-recognized evaluation system, there is some controversy over HER2 clinical expression status in OSCC or HNSCC, limiting the application of HER2 or pan HER2 target therapy in this region [32–34].

Compared to the other HER2 testing methods, IHC semi-quantitative testing is more available and much cheaper, and is also recommended by the American society of clinical oncology/college [35]. H score is one of the most commonly used IHC semi-quantitative methods to evaluate the target protein expression level, such as EGFR or HER2, and has been applied in OSCC [21, 36]. Hence this study employed a modified H score to measure the HER2 expression in OSCC, and revealed HER2 was expressed dramatically higher in the primary tumor than the normal mucosa, which was consistent with many previously published studies [37, 38]. In regards to the selection bias for advanced patients, the current cohort was of an integrated survival outcome with a much shorter follow up period, and found that HER2 high expression was positively correlated with poor OS and DFS, indicating HER2 as a promising prognostic predictor and molecular target in OSCC. In accordance with our study, some other relevant researches also indicated that HER2 overexpression might promote the invasion of OSCC and was positive related to the advanced tumor stage [37, 39]. It implied that HER2 might be a bio-mark for the biological behavior of OSCC and could anticipate the prognosis.

HNSCC patients with HER2 overexpression may be benefit from the dual anti-HER2/EGFR target therapy [40]. As an EGFR/HER2 dual inhibitor it is, PYR could partially overcome EGFR resistance [18]. Through aseries of in vitro and in vivo experiments, PYR was found to have a robust efficiency in suppressing the growth of OSCC cell lines and xenograft tumors in a concentration-dependent manner rather than a time dependent manner, especially in the low andintermediate concentration. This suggests that PYR might gain a fast response on OSCC and would be a candidate for neoadjuvant therapy [41, 42]. As indicated by some other studies, our study also found PYR induced cell apoptosis and G0-G1 cell cycle arrest, which meant thecombined application of PYR and chemotherapy might provide morebeneficial to OSCC patients [43, 44].

The PI3K/AKT and MAPK cascades were the main downstream of HER2 and EGFR [45]. The cross talk between the two cascades was regarded as the main mechanism of resistance to EGFR target therapy [12, 40]. Zhao TC et al. indicated growth differentiation factor-15 overexpression promoted OSCC by binding to HER2 and an HER2 phosphorylation inhibitor could suppress OSCC by blocking the phosphorylation of HER2, as well as AKT and ERK in its downstream [46]. In the present study, western blotting revealed that the phosphorylation, but not the expression levels, of HER2, AKT and ERK, were dramatically downregulated in the CAL27 cell line and the xenograft tumors by PYR. This result was consistent with the previous studies of PYR on other tumors, and suggests the potential mechanism of its anti-tumor effect [47, 48].

The current standard therapy for locally advanced OSCC usually loses control of the disease. The HER2 high expression predicts a poor prognosis with high recurrence and mortality, calling for novel systemic therapy. Pan HER2 inhibitors, such as PYR, may provide additional benefits, especially in advanced OSCC patients. Of course, further preclinical studies and clinical trials are needed to verify our findings.

## Conclusions

This is the first study to exhibit the anti-tumor effects of PYR on OSCC in vitro and in vivo, and demonstrated PYR inhibited OSCC cells by inducing apoptosis via the PI3K/ AKT and MAPK pathway.

### Electronic supplementary material

Below is the link to the electronic supplementary material.

Supplementary Material 1

## Data Availability

The datasets used and/or analyzed during the present study are available from the corresponding author on reasonable request.

## Abbreviations

HER2: Human epidermal growth factor receptor 2
EGFR: Epidermal growth factor receptor
OSCC: Oral squamous cell carcinoma
PYR: Pyrotinib
TMA: Tissue microarray
HNSCC: Head neck squamous cell carcinoma
TKI: Tyrosine kinase inhibitor
FDA: food drug administration
NCCN: National Cancer Comprehensive Network
IHC: Immunohistochemical
OS: overall survival
DFS: disease free survival
MAPK: mitogen-activating protein kinase
NSG: NOD-SCID gamma
HR: Hazard ratio
H&E: Hematoxylin-eosin
CCK8: Cell counting kit-8
CST: Cell signaling technology
