# Supplementary material for: The efficacy and potential mechanisms of pyrotinib in targeting EGFR and HER2 in advanced oral squamous cell carcinoma
Source: BMC Oral Health. 2024 Aug 6;24:898. doi: 10.1186/s12903-024-04459-4 (PMC11302363; doi:10.1186/s12903-024-04459-4)
Supplement: Supplementary file 1 — Supplementary Material 1 [file 12903_2024_4459_MOESM1_ESM.docx]

**Supplementary File**

**The Efficacy and Potential Mechanisms of Pyrotinib in Targeting EGFR and HER2 in Advanced Oral Squamous Cell Carcinoma**


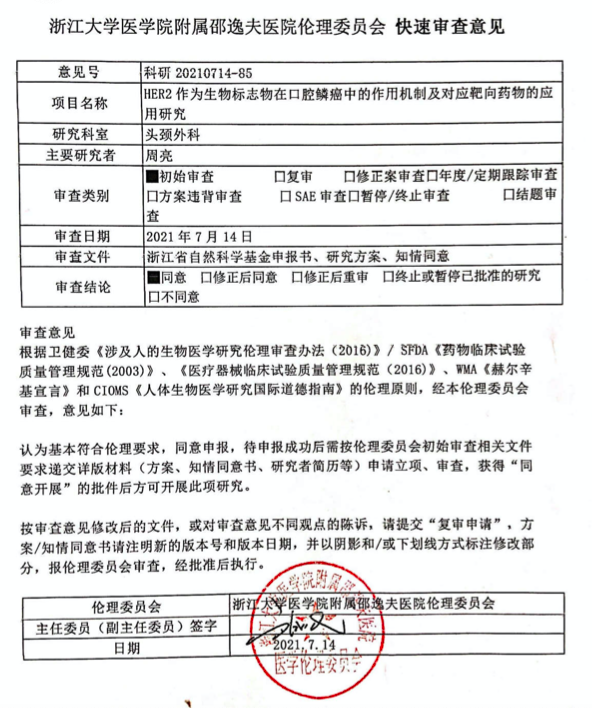


**Figure S1** the approval of ethics Committee of Sir Run Run Shaw hospital for the clinical samples and data.


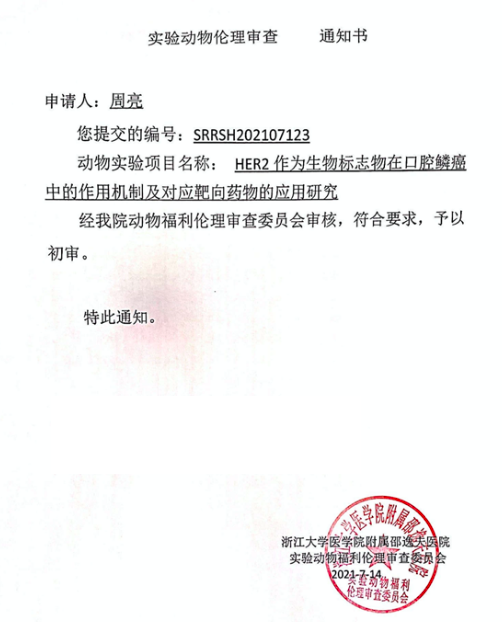


**Figure S2** the approval of the Animal ethics Committee of Sir Run Run Shaw hospital for the animal experiments

**Account for Western blotting**

In this study, WB performed film exposure using Thermo’s rainbow markers. The markers were visible during the transfer process, but not during the exposure process. All of the strips shown in this article are original images.


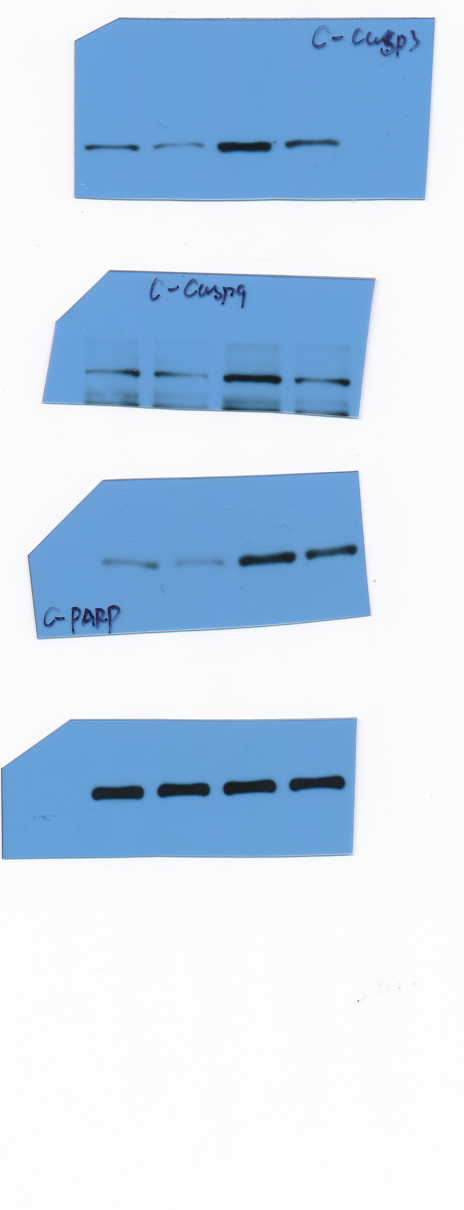


**Figure S3.** The original blot of the Figure 3F

The expression levels of proteins related to the apoptosis signaling in CAL27 and HN30 after the treatment of vehicles and pyrotinib for 24h. Starting from the left, there were the negative control of CAL27 (first) and HN30 (second), and the pyrotinib groups of CAL27 (third) and HN30 (fourth).


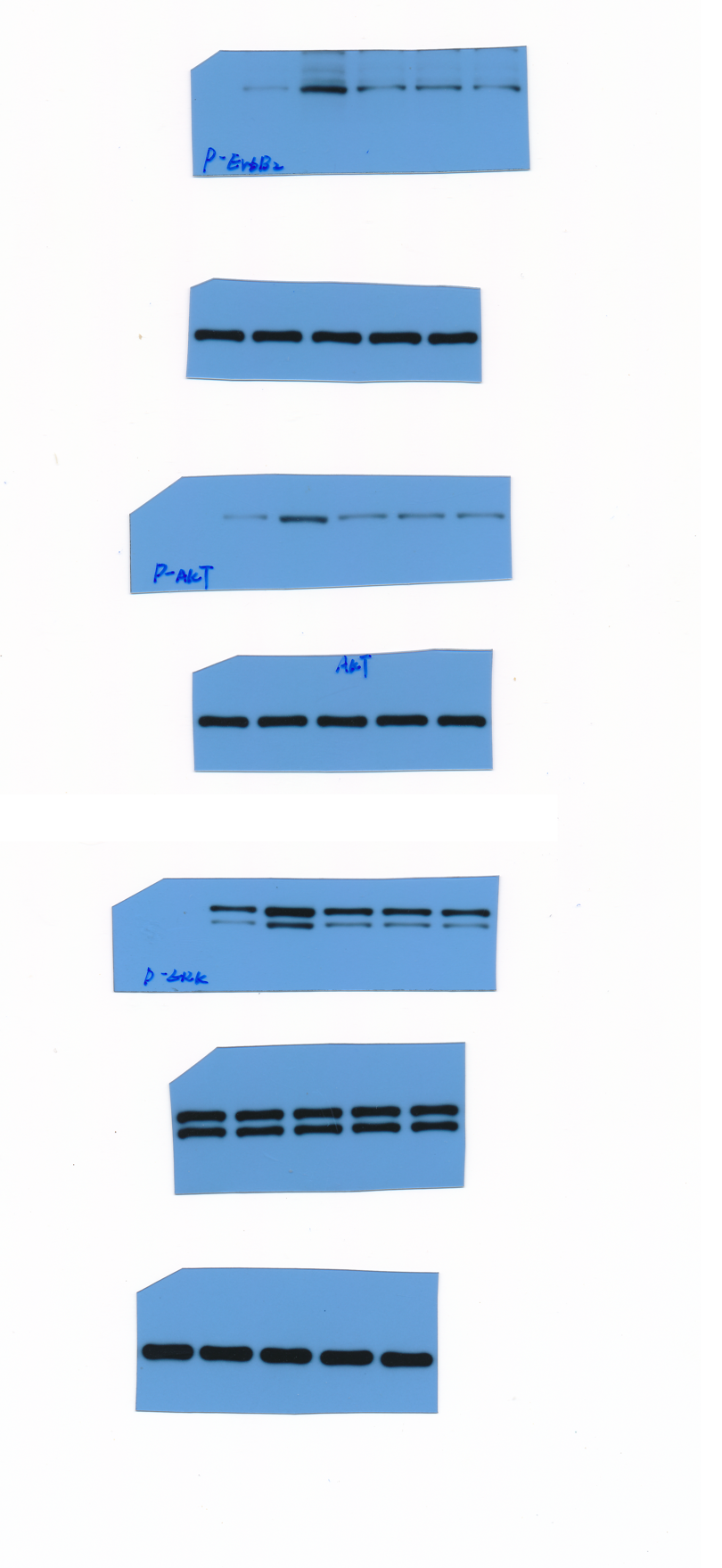


**Figure S4A.** The original blot of figure 4F

The expression levels and phosphorylation levels of the key proteins in the PI3K/AKT and MAPK signaling pathway after the treatment of gradient concentration of pyrotinib in CAL27 cell line.


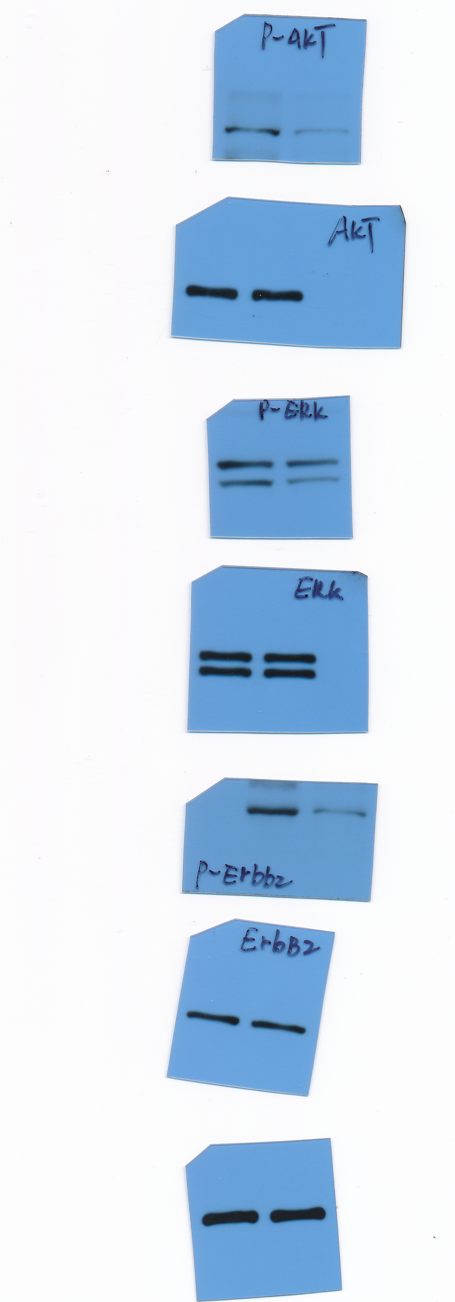


**Figure S4B.** The original blot of figure 4G

The expression levels and phosphorylation levels of the key proteins in the PI3K/AKT and MAPK signaling pathway after the treatment of vehicle and pyrotinib in CAL27 xenograft tumor. The left is the negative control.
